# Supplementary material for: From sequence to enzyme mechanism using multi-label machine learning
Source: BMC Bioinformatics. 2014 May 19;15:150. doi: 10.1186/1471-2105-15-150 (PMC4229970; doi:10.1186/1471-2105-15-150)
Supplement: Additional file 2 — Java code of ml2db. Additional file ml2db_code.tar.gz contains the Java source code to run the multi-label machine learning experiments and save the results to database. The code’s Javadoc is included. [file 1471-2105-15-150-S2.zip › additional file 2/ml2db/ml2db/doc/main/ml2db/al/utility/confidence/ConfidencesStorage.html]

ConfidencesStorage


JavaScript is disabled on your browser.


- Overview
- Package
- Class
- Use
- Tree
- Deprecated
- Index
- Help

- Prev Class
- Next Class

- Frames
- No Frames

- All Classes

- Summary:
- Nested |
- Field |
- Constr |
- Method

- Detail:
- Field |
- Constr |
- Method


main.ml2db.al.utility.confidence

## Class ConfidencesStorage

- java.lang.Object
- - main.ml2db.al.utility.confidence.ConfidencesStorage

- ---

    

  ```
  public class ConfidencesStorage
  extends java.lang.Object
  ```

- - ### Field Summary

    Fields

    | Modifier and Type | Field and Description |
    | --- | --- |
    | `java.util.HashMap<java.lang.String,java.lang.Double>` | `m_uncertainties` Stores for a set of confidences, its uncertainty (distance between average confidence over all labels and 50%) |
  - ### Constructor Summary

    Constructors

    Constructor and Description

    `ConfidencesStorage()`

    `ConfidencesStorage(int hashInitialCapacity)`
  - ### Method Summary

    Methods

    | Modifier and Type | Method and Description |
    | --- | --- |
    | `java.lang.Double` | `find(double[] confidences)` |
    | `java.util.HashMap<java.lang.String,java.lang.Double>` | `getMap()` |

    - ### Methods inherited from class java.lang.Object

      `equals, getClass, hashCode, notify, notifyAll, toString, wait, wait, wait`

- - ### Field Detail


    - #### m\_uncertainties

      ```
      public java.util.HashMap<java.lang.String,java.lang.Double> m_uncertainties
      ```

      Stores for a set of confidences, its uncertainty (distance between
      average confidence over all labels and 50%)
  - ### Constructor Detail


    - #### ConfidencesStorage

      ```
      public ConfidencesStorage()
      ```


    - #### ConfidencesStorage

      ```
      public ConfidencesStorage(int hashInitialCapacity)
      ```
  - ### Method Detail


    - #### find

      ```
      public java.lang.Double find(double[] confidences)
      ```


    - #### getMap

      ```
      public java.util.HashMap<java.lang.String,java.lang.Double> getMap()
      ```


- Overview
- Package
- Class
- Use
- Tree
- Deprecated
- Index
- Help

- Prev Class
- Next Class

- Frames
- No Frames

- All Classes

- Summary:
- Nested |
- Field |
- Constr |
- Method

- Detail:
- Field |
- Constr |
- Method
